# Electromyographic comparison of infraspinatus, anterior, and posterior deltoid fibres during belly press exercise and triceps overactivity

**DOI:** 10.1016/j.asmart.2025.08.004

**Published:** 2025-09-04

**Authors:** Yoshifumi Nanba, Tatsuyuki Ohta, Teruhiko Takata

**Affiliations:** a Department of Physical Therapy, Faculty of Rehabilitation, Kobe International University, Kobe, Japan; b Department of Rehabilitation, Seijin-kai Okubo Hospital, Akashi, Japan

**Keywords:** Belly press exercise, Dynamic stabilization, Electromyography, Posterior deltoid, Triceps brachii overactivity

## Abstract

**Background/objective:**

The Belly Press Exercise (BPE) is a key clinical tool, but its efficacy is dependent on proper execution. This study aimed to clarify the muscle activation patterns that differentiate a proper BPE from an inappropriate pattern characterized by compensatory shoulder extension. We hypothesized that an appropriate pattern would involve greater posterior deltoid activation for stabilization, while the inappropriate pattern would show increased triceps brachii activity.

**Methods:**

Surface electromyography was used to record the activity of the infraspinatus, anterior deltoid, posterior deltoid, and long head of the triceps brachii in 15 healthy male participants. The sample size was based on prior similar investigations (e.g., [13, 19]). Muscle activation was compared between two conditions: an appropriate pattern (isolated internal rotation) and an inappropriate pattern (internal rotation with 10° of shoulder extension) under three different load conditions (2, 5, and 10 % of body weight).

**Results:**

Posterior deltoid activity was significantly higher in the appropriate pattern across all load conditions. In contrast, triceps brachii activity was significantly higher in the inappropriate pattern, particularly under the 5 % and 10 % load conditions. No significant differences were observed for the anterior deltoid or infraspinatus between patterns.

**Conclusion:**

The posterior deltoid acts as a key stabilizer during a proper BPE, while overactivity of the long head of the triceps brachii is a clear indicator of compensatory shoulder extension. These findings provide clinicians with evidence-based markers for monitoring BPE form to ensure its therapeutic efficacy and safety.

## 1 Introduction

**Fig. 1:**
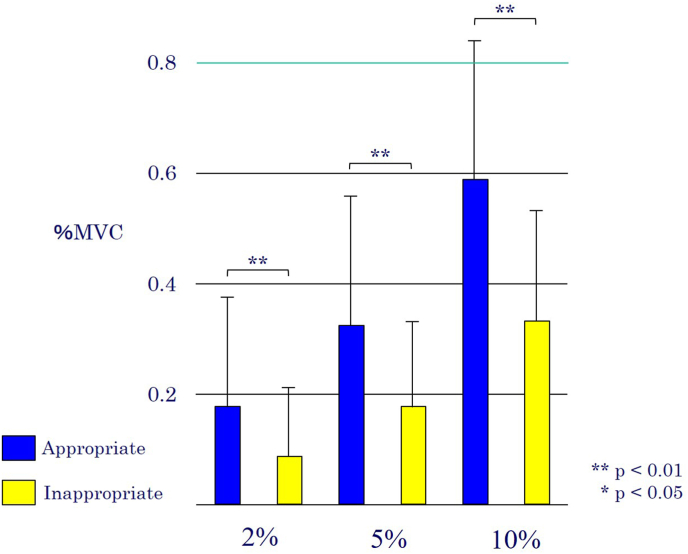
Deltoid posterior (Mean of %MVC).

**Fig. 2:**
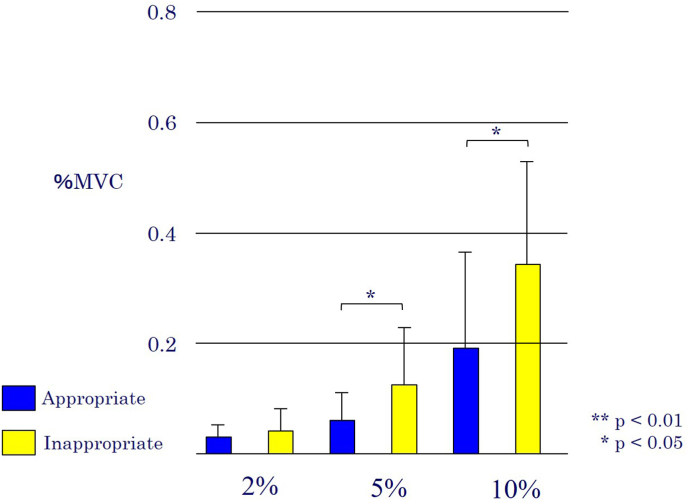
Triceps long head (mean of %MVC).

The Belly Press Exercise (BPE) is a widely used clinical tool for both the assessment and strengthening of the subscapularis muscle, a key internal rotator of the shoulder. Proper execution of the BPE is critical for accurate diagnosis of subscapularis tears and for effective rehabilitation, as improper form can lead to inaccurate assessments and place undue stress on the anterior joint capsule, a significant concern in postoperative patients. One common compensatory movement is unintentional shoulder extension, which may alter the activation patterns of stabilizing and prime mover muscles. To maintain glenohumeral stability during BPE, synergistic activation of other shoulder muscles is required. The posterior deltoid, for example, is thought to play a crucial role in "shoulder muscle stabilization" by counteracting the anteriorly directed forces on the humeral head during internal rotation, thus preventing impingement and instability. Conversely, the long head of the triceps brachii, a primary shoulder extensor, may become overactive during an improper BPE, contributing to the compensatory extension movement. The infraspinatus and anterior deltoid are also involved in the complex balance of forces around the shoulder during this exercise. Understanding the interplay of these muscles is essential for optimizing BPE technique (see Fig. 1, Fig. 2).

However, the specific muscle activation patterns that differentiate a proper BPE from an inappropriate, compensatory pattern have not been fully elucidated. Therefore, the purpose of the present study was to compare the electromyographic (EMG) activity of the infraspinatus, anterior deltoid, posterior deltoid, and long head of the triceps brachii during BPE under two conditions: (1) an appropriate pattern and (2) an inappropriate pattern characterized by compensatory shoulder extension. We hypothesized that the appropriate pattern would elicit greater posterior deltoid activation than the inappropriate pattern, reflecting its stabilizing role. We further hypothesized that the inappropriate pattern would be characterized by significantly higher activation of the long head of the triceps brachii.

## 2 Methods

The sample size of 15 participants was determined based on previous, similar EMG studies of the shoulder (e.g., Refs. ^1^,^2^). Participants were seated in a chair with a backrest. BPE involves positioning the humerus in a dependent position, bending the elbow at approximately 90°, and moving the palm towards the abdominal midline. Two distinct movement patterns were analyzed: an appropriate pattern, defined as isolated shoulder internal rotation, performed while maintaining 90° of elbow flexion and without concomitant shoulder extension or flexion; and an inappropriate pattern with approximately 10° of shoulder extension accompanying internal rotation. This angle was chosen as it represents a common compensatory movement observed clinically, and it was monitored using a standard goniometer. Resistance was measured using a hand dynamometer (OG Giken ISOFORCE, Okayama, Japan).

EMG data were collected from the left anterior deltoid, posterior deltoid, infraspinatus, and long head of the triceps brachii using surface electrodes, following the SENIAM guidelines. To determine the maximum voluntary contraction (MVC), subjects performed a 10-s maximal isometric contraction for each muscle according to the method described by Daniels et al. Following the methodology of Yang & Winter,^3^ we analyzed the central 8-s portion of the 10-s trial to exclude the initial ramp-up and terminal fatigue phases. The peak 1-s root mean square (RMS) value within this central portion was identified, and this peak value was taken as the MVC. All subsequent EMG data from the BPE trials were normalized to this MVC value (%MVC).

Statistical analyses were performed using R (version 4.3.2, R Foundation for Statistical Computing, Vienna, Austria). A two-way repeated measures ANOVA was used to examine the effects of execution pattern (appropriate vs. inappropriate) and load condition (2 %, 5 %, 10 % BW) on muscle activity for each muscle. The significance level was set at p < 0.05.

## 3 Results

A two-way repeated measures ANOVA revealed a significant interaction effect between execution pattern and load condition for the posterior deltoid (p = .004) and the long head of the triceps brachii (p = .002). For the posterior deltoid, post-hoc analysis showed that activation was significantly higher in the appropriate pattern compared to the inappropriate pattern under all load conditions (2 %, 5 %, and 10 %). Conversely, for the long head of the triceps brachii, activation was significantly higher in the inappropriate pattern at the 5 % and 10 % load conditions. No significant main effect for execution pattern was found for the anterior deltoid or the infraspinatus (p = .129 and p = .572, respectively).

A significant main effect of load was observed, demonstrating a progressive increase in muscle activation as load increased. Comparing the 10 % load to the 2 % load within the appropriate pattern, activation of the anterior deltoid increased 4.40-fold, posterior deltoid 3.32-fold, infraspinatus 2.44-fold, and the long head of the triceps brachii 6.19-fold. This load-dependent response was observed across all measured muscles (Table 1, Table 2, Table 3, Table 4).

**Table 1:** Anterior deltoid fibers.

2 %			5 %		10 %	
Appropriate		ERROR	Appropriate	ERROR	Appropriate	ERROR
**0.090**		**0.080**	**0.160**	**0.140**	**0.310**	**0.330**
**0.030**		**0.030**	**0.030**	**0.030**	**0.040**	**0.040**
**0.130**		**0.060**	**0.160**	**0.050**	**0.220**	**0.070**
**0.088**		**0.071**	**0.910**	**0.069**	**0.189**	**0.102**
**0.027**		**0.022**	**0.035**	**0.045**	**0.063**	**0.098**
**0.010**		**0.010**	**0.020**	**0.010**	**0.020**	**0.020**
**0.117**		**0.117**	**0.238**	**0.093**	**0.842**	**0.214**
**0.049**		**0.261**	**0.058**	**0.242**	**0.164**	**0.564**
**0.004**		**0.030**	**0.010**	**0.010**	**0.160**	**0.120**
**0.090**		**0.020**	**0.070**	**0.020**	**0.280**	**0.080**
**0.040**		**0.010**	**0.040**	**0.020**	**0.090**	**0.050**
**0.020**		**0.001**	**0.010**	**0.010**	**0.020**	**0.020**
**0.070**		**0.030**	**0.130**	**0.030**	**0.400**	**0.090**
**0.020**		**0.020**	**0.030**	**0.020**	**0.070**	**0.050**
**0.090**		**0.010**	**0.160**	**0.020**	**0.500**	**0.120**

**AVG**	**0.058**	**0.051**	**0.137**	**0.054**	**0.225**	**0.131**
**SD**	**0.039**	**0.064**	**0.217**	**0.061**	**0.215**	**0.139**

**p-value**	**n.s**		**n.s**		**n.s**	

∗p < .05 ∗∗p < .01.

**Table 2:** Posterior deltoid fibers.

%BW	2 %		5 %		10 %	
No.	Appropriate	ERROR	Appropriate	ERROR	Appropriate	ERROR
**1**	**0.090**	**0.070**	**0.410**	**0.160**	**0.930**	**0.520**
**2**	**0.010**	**0.010**	**0.030**	**0.020**	**0.160**	**0.100**
**3**	**0.220**	**0.060**	**0.300**	**0.130**	**0.470**	**0.230**
**4**	**0.131**	**0.064**	**0.437**	**0.036**	**0.631**	**0.102**
**5**	**0.660**	**0.054**	**0.144**	**0.296**	**0.642**	**0.749**
**6**	**0.020**	**0.010**	**0.080**	**0.050**	**0.190**	**0.180**
**7**	**0.330**	**0.071**	**0.336**	**0.072**	**0.593**	**0.225**
**8**	**0.099**	**0.261**	**0.215**	**0.421**	**0.968**	**0.270**
**9**	**0.030**	**0.009**	**0.181**	**0.030**	**0.327**	**0.161**
**10**	**0.080**	**0.050**	**0.150**	**0.050**	**0.510**	**0.260**
**11**	**0.550**	**0.470**	**0.800**	**0.620**	**0.920**	**0.454**
**12**	**0.080**	**0.020**	**0.270**	**0.090**	**0.710**	**0.560**
**13**	**0.040**	**0.020**	**0.160**	**0.160**	**0.380**	**0.540**
**14**	**0.140**	**0.040**	**0.330**	**0.090**	**0.810**	**0.460**
**15**	**0.201**	**0.099**	**0.614**	**0.427**	**0.340**	**0.190**

**AVG**	**0.177**	**0.087**	**0.275**	**0.177**	**0.589**	**0.333**
**SD**	**0.194**	**0.123**	**0.186**	**0.167**	**0.255**	**0.193**

**p-value**	**∗**		**∗∗**		**∗∗**	

∗p < .05 ∗∗p < .01.

**Table 3:** Infraspinatus.

2 %			5 %		10 %	
Appropriate		ERROR	Appropriate	ERROR	Appropriate	ERROR
**0.160**		**0.130**	**0.220**	**0.210**	**0.510**	**0.460**
**0.170**		**0.250**	**0.270**	**0.340**	**0.380**	**0.670**
**0.110**		**0.100**	**0.140**	**0.140**	**0.240**	**0.240**
**0.221**		**0.317**	**0.290**	**0.592**	**0.476**	**""**
**0.069**		**0.078**	**0.081**	**0.129**	**0.120**	**0.165**
**0.060**		**0.050**	**0.090**	**0.050**	**0.130**	**0.180**
**0.623**		**0.625**	**0.818**	**0.489**	**""**	**0.748**
**0.195**		**0.961**	**0.241**	**""**	**0.534**	**""**
**0.038**		**0.033**	**0.118**	**0.094**	**0.288**	**0.311**
**0.100**		**0.090**	**0.130**	**0.100**	**0.280**	**0.180**
**0.080**		**0.060**	**0.180**	**0.160**	**0.44 0**	**0.480**
**0.060**		**0.050**	**0.110**	**0.120**	**0.400**	**0.220**
**0.160**		**0.090**	**0.340**	**0.170**	**0.650**	**0.540**
**0.030**		**0.040**	**0.070**	**0.160**	**0.120**	**0.390**
**0.080**		**0.090**	**0.150**	**0.130**	**0.360**	**0.400**

**AVG**	**0.144**	**0.198**	**0.217**	**0.206**	**0.352**	**0.383**
**SD**	**0.140**	**0.253**	**0.179**	**0.152**	**0.159**	**0.184**

**p-value**	**n.s**		**n.s**		**n.s**	

∗p < .05 ∗∗p < .01.

**Table 4:** Long head of the triceps brachii.

2 %			5 %		10 %	
Appropriate		ERROR	Appropriate	ERROR	Appropriate	ERROR
**0.040**		**0.060**	**0.110**	**0.240**	**0.510**	**0.690**
**0.060**		**0.070**	**0.080**	**0.100**	**0.160**	**0.280**
**0.070**		**0.060**	**0.080**	**0.090**	**0.150**	**0.250**
**0.038**		**0.035**	**0.048**	**0.040**	**0.090**	**0.328**
**0.035**		**0.026**	**0.038**	**0.227**	**0.064**	**0.411**
**0.009**		**0.010**	**0.040**	**0.050**	**0.130**	**0.180**
**0.071**		**0.102**	**0.230**	**0.104**	**0.689**	**0.497**
**0.038**		**0.142**	**0.041**	**0.394**	**0.176**	**""**
**0.009**		**0.009**	**0.023**	**0.021**	**0.074**	**0.128**
**0.020**		**0.030**	**0.030**	**0.060**	**0.100**	**0.200**
**0.010**		**0.040**	**0.060**	**0.300**	**0.160**	**0.190**
**0.010**		**0.010**	**0.010**	**0.060**	**0.110**	**0.380**
**0.020**		**0.020**	**0.090**	**0.040**	**0.320**	**0.340**
**0.010**		**0.010**	**0.010**	**0.050**	**0.060**	**0.240**
**0.020**		**0.010**	**0.030**	**0.120**	**0.090**	**0.760**

**AVG**	**0.031**	**0.042**	**0.061**	**0.126**	**0.192**	**0.348**
**SD**	**0.021**	**0.038**	**0.053**	**0.108**	**0.174**	**0.182**

**p-value**	**n.s**		**∗**		**∗**	

∗p < .05 ∗∗p < .01.

## 4 Discussion

The present study compared the muscle activation patterns of key shoulder muscles between appropriate and inappropriate execution of the Belly Press Exercise (BPE), confirming our primary hypotheses. Our finding that posterior deltoid activity was significantly higher during the appropriate pattern supports its crucial function as a dynamic stabilizer of the glenohumeral joint.^4^^,^^5^ This aligns with previous findings that highlight the importance of coordinated muscle activation for shoulder stability.^6^^,^^2^ By stabilizing the humeral head, the posterior deltoid allows for more isolated and effective activation of the target subscapularis muscle. Clinicians can therefore interpret heightened posterior deltoid activation as an indicator of correct BPE form.

Conversely, the significantly increased triceps activation during the inappropriate pattern highlights its role in the common compensatory strategy of shoulder extension.^7^ This is particularly relevant for postoperative patients where protecting the healing anterior capsule is paramount.^8^^,^^9^ When the subscapularis is weak or inhibited, patients may use the powerful long head of the triceps to extend the shoulder, creating the illusion of internal rotation. This compensatory action not only reduces the therapeutic effect on the subscapularis but can also place potentially harmful stress on anterior structures. Therefore, a key recommendation for clinicians is to monitor for and correct this specific compensation.

### 4.1 Limitations

Several limitations in this study should be acknowledged. First, our analysis was restricted to surface EMG, which cannot measure the activity of deep muscles like the subscapularis. Future studies using fine-wire EMG are warranted to investigate the subscapularis directly during these tasks. Second, our cohort consisted only of young, healthy male participants. This may limit the generalizability of our findings to other populations, such as female participants or patients with actual shoulder pathology, where muscle activation patterns may differ. Finally, while we standardized the inappropriate pattern based on common clinical observation, other forms of improper execution exist, and further research is needed to investigate these variations.

## 5 Conclusion

In conclusion, the activation of the posterior deltoid is a key feature of a proper BPE, serving a critical stabilizing role. In contrast, overactivity of the long head of the triceps brachii is a clear indicator of a common and potentially harmful compensatory movement involving shoulder extension. These findings provide clinicians with clear, evidence-based indicators for monitoring and correcting BPE form, thereby helping to ensure its efficacy and safety in shoulder rehabilitation settings.
